# Red Blood Cell Distribution Width Is Associated with Deterioration of Renal Function and Cardiovascular Morbidity and Mortality in Patients with Diabetic Kidney Disease

**DOI:** 10.3390/life10110301

**Published:** 2020-11-22

**Authors:** Stefanos Roumeliotis, Aikaterini Stamou, Athanasios Roumeliotis, Marios Theodoridis, Konstantinos Leivaditis, Stylianos Panagoutsos, Vassilios Liakopoulos

**Affiliations:** 1 Division of Nephrology and Hypertension, 1st Department of Internal Medicine, AHEPA Hospital, School of Medicine, Aristotle University of Thessaloniki, 54636 Thessaloniki, Greece; aroumeliotis@auth.gr (A.R.); konleiv@windowslive.com (K.L.); liakopul@otenet.gr (V.L.); 2 Department of Microbiology, AHEPA Hospital, School of Medicine, Aristotle University of Thessaloniki, 54636 Thessaloniki, Greece; katerina_stms@yahoo.gr; 3 Department of Nephrology, School of Medicine, Democritus University of Thrace, 68100 Alexandroupolis, Greece; cymarth@otenet.gr (M.T.); spanagou@med.duth.gr (S.P.)

**Keywords:** red blood distribution width, type 2 diabetes mellitus, diabetic kidney disease, cardiovascular events, mortality

## Abstract

We sought to investigate the possible association between Red Blood Cell Distribution Width (RDW), vascular calcification, oxidative stress and renal function and all-cause/cardiovascular (CV) mortality, CV events and progression of kidney disease in a cohort of patients with Diabetic Kidney Disease (DKD). Carotid intima media thickness (cIMT) and oxidized low-density cholesterol were measured in 104 Type 2 Diabetes Mellitus (T2DM) patients with established DKD, distributed in all five stages of kidney disease and 38 diabetics with normal renal function. All patients were followed for 7 years with end-points all-cause and CV mortality, CV events and progression to End-Stage Renal Disease (ESRD). RDW was positively correlated with diabetes duration (*r* = 0.19, *p* = 0.023) and albuminuria (*r* = 0.29, *p* = 0.002). Multivariate regression analysis revealed that RDW was a strong, independent predictor of cIMT value (β = 0.031, *p* = 0.012). Kaplan-Meier curves and Cox proportional hazard models revealed that after adjustment for several cofounders, RDW was a significant and independent predictor for all-cause mortality, CV mortality, CV event and progression to ESRD (HR 1.75, *p* = 0.001, HR 2.03, *p* = 0.001, HR = 1.66, *p* < 0.0001 and HR 2.14, *p* = 0.001 respectively). RDW predicts mortality, CV events and deterioration of renal function in DKD, probably reflecting atherosclerosis.

## 1. Introduction

Red Blood Cell Distribution Width (RDW) is an index of Red Blood Cell (RBC) size variability (anisocytosis) and is routinely included in the complete blood cell count [1]. Until recently, RDW has been used for differential diagnosis of types of anemias [2]. Higher RDW values indicate shortened erythrocyte life span due to poor erythropoiesis or enhanced RBC destruction. During the past decade, there is a growing body of evidence that high RDW is an independent predictor of cardiovascular (CV) morbidity and mortality in the general population [3], chronic heart failure [4], coronary heart disease [5], stroke [6], kidney transplant recipients [7] and acute kidney injury (AKI) treated with hemodialysis (HD) [8]. Additionally, it has been reported that high RDW is associated with the presence of Type 2 diabetes mellitus (T2DM), the progression of chronic kidney disease (CKD) in patients with different degrees of renal impairment [9] and with albuminuria in the general population [10]. The underlying mechanisms linking high RDW and all these severe conditions are yet unclear, but chronic inflammation, endothelial dysfunction, oxidative stress (OS) and nutritional deficiencies have been proposed as possible etiologies. CKD and T2DM are conditions characterized by chronic subclinical inflammation, OS and endothelial injury [11] with consequent accelerated atherosclerosis, deterioration of renal function and increased CV morbidity [12]. Due to the severe CV risk burden in this population, there is recent interest in the identification of novel biomarkers that can predict mortality, CV events and disease progression in patients with diabetic kidney disease (DKD). Although RDW has been widely investigated in various populations, data regarding the possible predictive role of RDW for CV morbidity and mortality and deterioration of renal function in DKD is limited. Moreover, although there is evidence supporting that RDW is tightly associated with endothelial dysfunction in CKD patients [9], this has not been fully investigated in DKD patients. Furthermore, there is no data regarding the association between RDW and OS.

Therefore, we sought to investigate the association between RDW and all-cause CV mortality, CV events and progression of CKD in a cohort of patients with DKD. Moreover, we further aimed to explore if the association between elevated RDW and increased risk for adverse events was due to the fact that anisocytosis reflected enhanced vascular calcification-assessed by carotid intima-media thickness (cIMT)- or OS, assessed by circulating oxidized-low density lipoprotein (ox-LDL) levels.

## 2. Results

Anthropometric, clinical and biochemical parameters of patients with T2DM according to median RDW are presented in Table 1. Patients in the high RDW group had significantly higher BMI, lower hemoglobin, higher C-reactive protein (CRP) and longer period of T2DM diagnosis. They also had higher urea and creatinine levels, lower estimated glomerular filtration rate (eGFR) and significantly higher proteinuria and albuminuria levels. Compared to the low, patients in the high RDW group presented significantly higher cIMT values. There was no difference among groups regarding CV events at baseline or previous, sex, anti-diabetic, anti-hypertensive medication and erythropoietin stimulating agents.

Table 2 shows the correlation matrix analysis of RDW with various anthropometric, clinical, nutritional, inflammatory and DKD markers. RDW correlated inversely with hemoglobin (*r* = −0.41, *p* < 0.0001), albumin (*r* = −0.20, *p* = 0.026) and eGFR (*r* = −0.33, *p* < 0.0001). There was a significant positive correlation between RDW and duration of T2DM (*r* = 0.19, *p* = 0.023), diastolic blood pressure (*r* = 0.18, *p* = 0.03), CRP (*r* = 0.24, *p* = 0.004), proteinuria (*r* = 0.30, *p* = 0.001) and albuminuria (*r* = 0.29, *p* = 0.002). Systolic blood pressure and body mass index (BMI) were positively correlated with RDW, but the association was marginally not significant.

**Figure 1:**
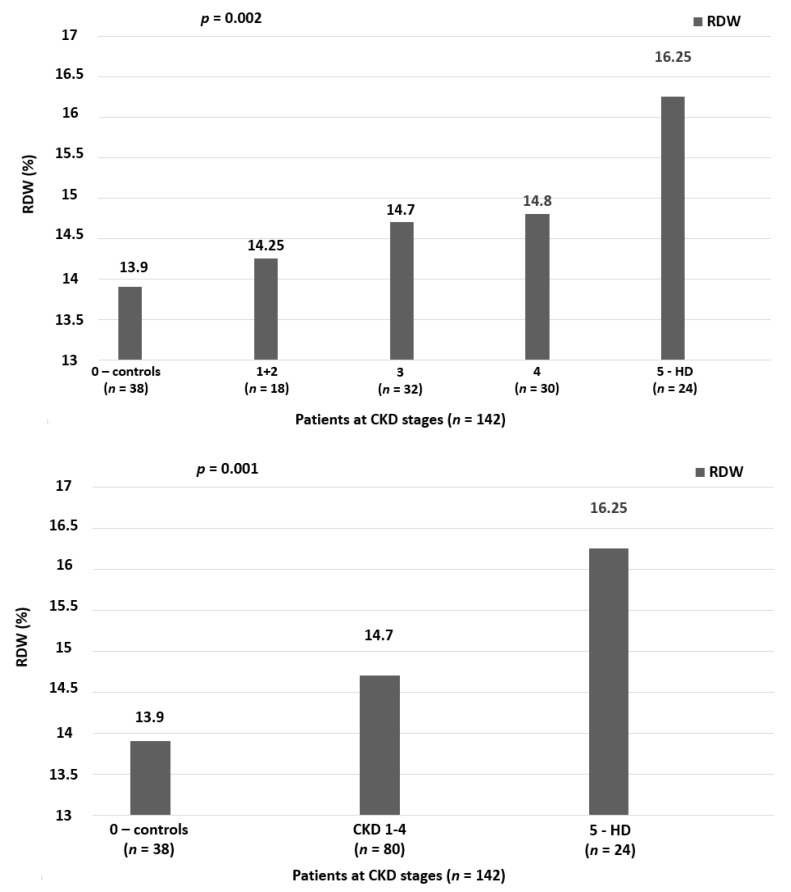
RDW in different stages of DKD.

RDW values according to stages of DKD are presented in Figure 1. Compared to diabetics with normal kidney function (stage 0), patients in early stage of CKD (stages 1 + 2) had significantly higher values of RDW (13.9% vs. 14.25%). RDW augmented significantly along with DKD progression, with the highest values presented in HD patients (16.25%). When the RDW values in controls, CKD and HD are presented separately, the difference of RDW among groups is even more statistically significant (*p* = 0.001). Although the normal range for RDW is 12–14%, we observed in our study that the controls had a RWD close to the upper limit (13.9%). This can be attributed to the fact that the control group included patients with T2DM for at least 10 years and the majority of those had hypertension. Both diabetes and hypertension are conditions predisposing to elevation of RDW. No association was observed between RDW and lipid profile parameters, including ox-LDL. RDW was significantly and positively correlated with cIMT (*r* = 0.22, *p* = 0.008).

Multiple regression analysis in Table 3 shows that in unadjusted model, only age was associated with cIMT. After adjustment for various well-established cofounders affecting cIMT value (age, sex, BMI, hemoglobin, albumin, total cholesterol, triglycerides, diastolic/systolic blood pressure, duration of T2DM, Glycated hemoglobin (HbA1c), CRP, eGFR, albuminuria and proteinuria), RDW was found to be the strongest independent factor predicting cIMT value.

Multiple regression analysis in Table 4 shows that after adjustment for the variables associated with RDW (diastolic blood pressure, duration of T2DM, serum albumin, CRP and eGFR), cIMT, hemoglobin and urine protein to creatinine ratio-UPCR were strong predictors of RDW. In our multiple regression analyses, even after adjusting for HD treatment (Supplementary Tables S1 and S2), our results remained practically the same.

Median time since diagnosis at baseline was 57.5 months (range 7 to 84 months). During the follow-up period, the mortality rate was 7.60 cases per 100-person years and the incidence rates for CV death and CV event were 5.35 and 13.15 cases per 100 patient years respectively. Thirty-one patients (42.5%) died in the high RDW group and 13 patients (18.8%) died in the low RDW group (*p* = 0.002). The leading cause of mortality in these 44 patients was CV (31/44, 70.5%). There was also a significant difference in CV death between the two groups, with 25 patients (34.2%) in the high RDW group and 6 patients (8.6%) in the low RDW group (*p* < 0.0001). During the study period, 66 CV events were documented (31 fatal/35 non-fatal), 43 (58.9%) in the high RDW group and 23 (33.3%) in the low RDW group (*p* = 0.002). Twenty-two of the non-dialysis patients progressed to end-stage renal disease (ESRD) (22/118, 15.5%), 18 (31.6%) in the high RDW group and 4 (6.5%), in the low group (*p* < 0.0001).

**Figure 2:**
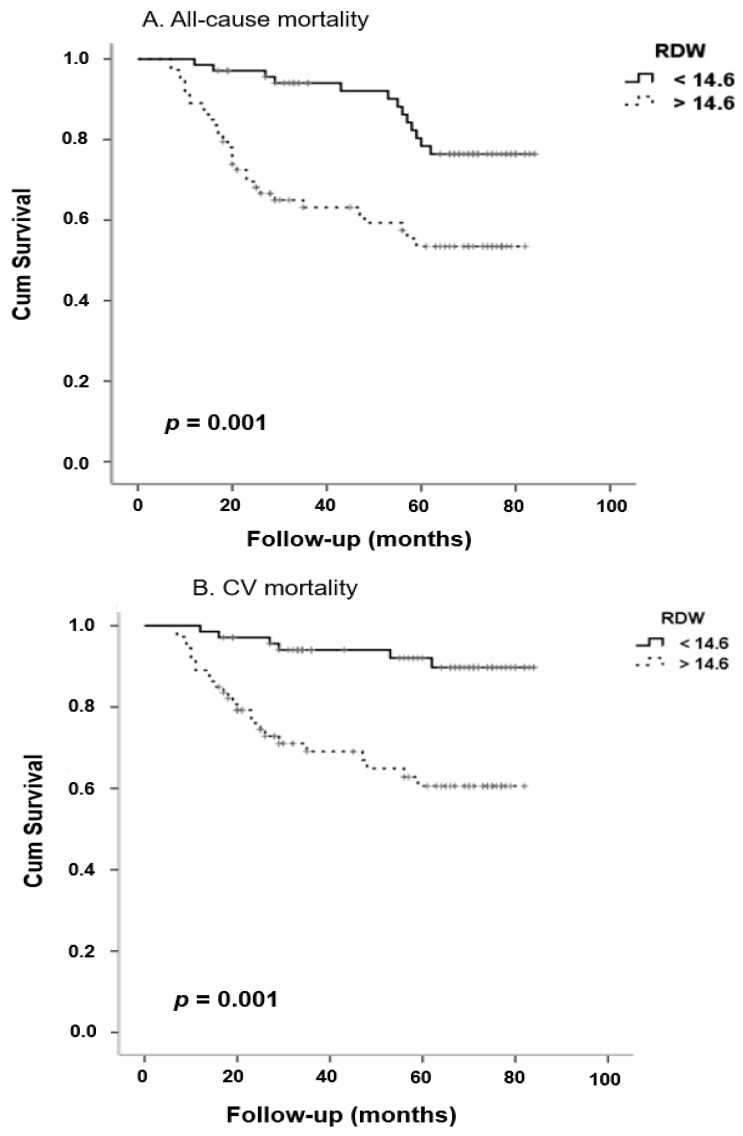

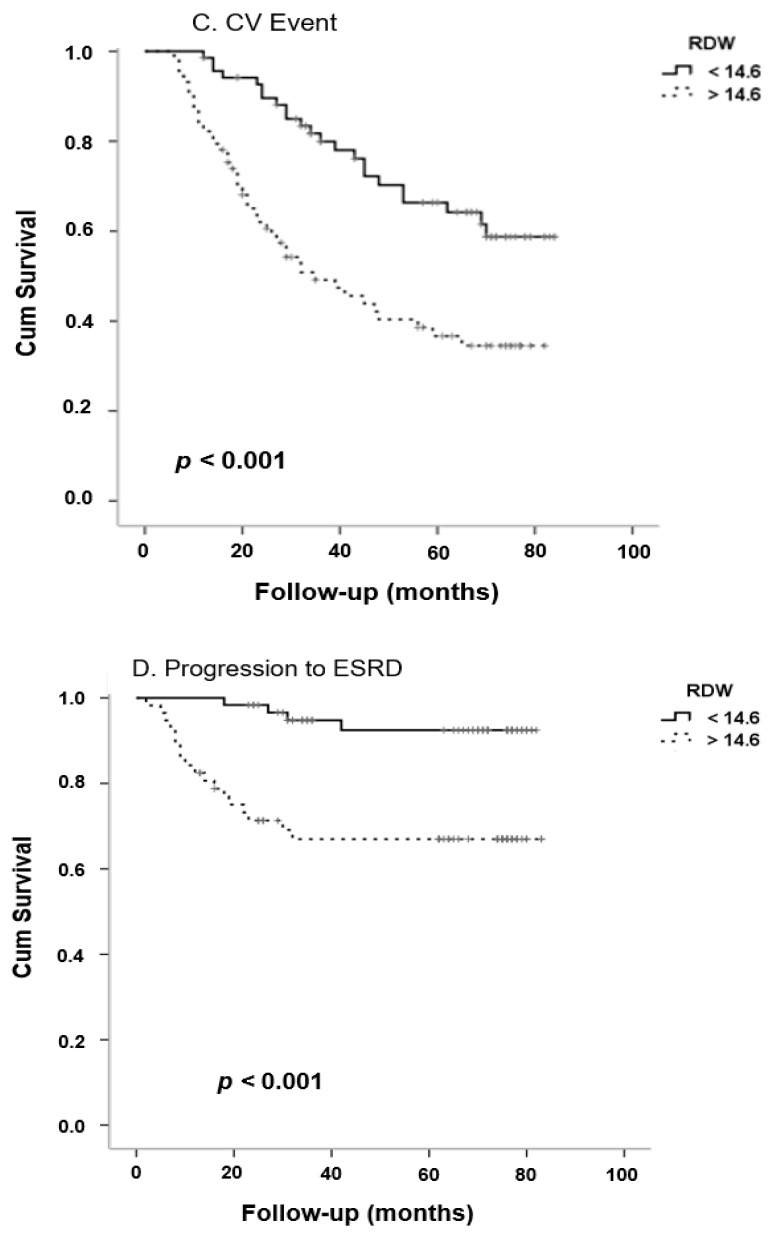
Kaplan-Meier curves for all-cause mortality (**A**), CV mortality (**B**), CV events (**C**) and progression to ESRD (**D**) in patients with high and low RDW levels (according to median value 14.6%). Log-rank test *p* = 0.001, <0.001 <0.001 and <0.001, respectively.

Kaplan-Meier analysis showed that patients with high RDW levels presented a significantly higher risk for all-cause mortality, CV mortality, CV event and progression to ESRD compared to the low RDW group (Figure 2; *p* = 0.001, *p* < 0.001, *p* < 0.001 and *p* < 0.001 respectively). After adjustment for several cofounding factors, Cox proportional hazard models revealed that RDW was a significant and independent predictor for all-cause mortality, CV mortality, CV event and progression to ESRD (Table 5, Hazard Ratio-HR 1.27, *p* = 0.04, HR 1.30, *p* = 0.01, HR = 1.40, *p* < 0.0001 and HR 1.36, *p* = 0.003 respectively).

## 3. Discussion

During the last decades RDW has emerged as a novel prognostic marker for serious adverse events such as all-cause and CV mortality in various populations [13]. However, the data regarding the predictive value of RDW and CV events and death in patients with various levels of renal impairment is still very scarce. The main finding of our study was that RDW is an independent predictor of DKD progression, all-cause and CV mortality in patients with established DKD.

Chronic inflammatory cell environment is a condition predisposing to enhanced OS and molecular apoptosis and therefore to subsequent enhanced RBC destruction [14]. In diabetics and CKD patients, several molecular pathways linking chronic inflammation and anisocytosis have been proposed: shortened erythrocyte lifespan, inhibited erythropoietin response and impaired iron metabolism [15,16]. Furthermore, it is well-established that T2DM and diabetic-related obesity are conditions characterized by chronic inflammation status [17]. In our study we found a strong linkage between RDW and CRP levels in DKD patients. In agreement with our results, RDW was found to be strongly correlated with high CRP levels in various populations: unselected subjects from the community [3,14], diabetics [18] and populations with various degrees of renal impairment such as -Peritoneal Dialysis (PD) [19], HD [20] and CKD [9].

Data from population-based studies showed clearly that RDW predicts mortality and adverse events in the general population. The National Health and Nutrition Examination Survey (NHANES) study, a large, community-based study showed that RDW was an independent predictor of all-cause and CV mortality in 15,852 adults from general population followed for 6 years [3]. The HRs for all-cause and CVD mortality were similar to those found in our study. The authors of the study hypothesized that strong linkage of RDW with increased inflammation status-assessed by increased CRP levels- might be the mechanism underlying this finding. In line with these results, several community-based studies reported the predictive role of RDW and adverse events-including death [21,22,23,24]. Malandrino et al., conducted a sub-analysis of the cross-sectional NHANES III study to investigate the possible predictive role of RDW for vascular complications, DKD and CV events in 2497 T2DM adults followed for 6 years [18]. Compared to diabetic adults on the lower, subjects on the higher RDW quartiles presented significantly higher risk for DKD, vascular complications and CV events. The authors concluded that RDW might be considered as a novel marker of T2DM duration, progression and a predictor of micro/macrovascular diabetic complications.

There is a growing body of evidence reporting that RDW predicts mortality, readmission to the hospital after major CV and other adverse events in various settings: coronary artery disease [25], congestive/acute and chronic heart failure [4,26,27,28,29,30], stroke [6], PAD [31], acute myocardial infarction [32,33], acute dyspnea [34] and severe sepsis [35]. A recent meta-analysis that included 28 studies and 102,689 patients with CV showed that RDW was strongly linked with all-cause mortality and major CV events [36]. However, until today, data suggesting that RDW is a strong predictor for mortality in CKD patients is scarce. The studies that investigated the possible association between RDW and all-cause/CV mortality and morbidity in subjects with different degrees of renal impairment are in agreement with our results. Hsieh et al. [37], conducted a retrospective observational study on 1075 patients with CKD stage 3–5, and found that RDW was independently associated with all-cause mortality, CV mortality and severe infection, with HRs similar to ours (all-cause mortality HR = 2.19, CV mortality HR = 2.28). Moreover, RDW was reported as a strong independent factor of all-cause mortality on populations with impaired renal function, such as patients with AKI that were treated with renal replacement therapy [8] and kidney transplant recipients [7]. In a meta-analysis of nine studies and 117,047 CKD patients (including HD and PD patients), Zhang et al. found that for every 1% increase in the RDW value, the risk of mortality was increased by 47% (HR 1.47, 95% Confidence Interval-CI 1.35–1.61), whereas in a subgroup analysis in HD patients, the risk of mortality was increased by 36% (HR 1.36, 95% CI 1.20–1.53) for every 1% increase in RDW [38].

Vashistha et al., performed an observational, retrospective, multicenter study in a large cohort of 109,675 chronic, stable patients undergoing HD and found a graded, strong association between high RDW and high all-cause mortality risk, regardless anemia parameters [20]. Similarly to our findings, a RDW value above 14.9% was significantly associated with all-cause and CV mortality in a cohort of 80 HD patients, followed for 5 years [39]. Moreover, in PD populations, high RDW was found to be a risk marker for several adverse events, including CV events, hospitalizations and mortality [40,41,42].

CIMT is a surrogate marker for subclinical atherosclerosis closely linked with CV events and mortality in general population and patients with DKD [43]. In our cohort of DKD patients there was a strong positive association between cIMT and RDW values. Furthermore, cIMT was found to be the strongest independent predictor of RDW values, compared to several parameters that had been repeatedly related with RDW values. In agreement with our results, there is accumulating data supporting the strong linkage between elevated RDW and subclinical atherosclerosis-assessed by advanced cIMT-in various settings. Data from large community-based epidemiologic studies showed that RDW was tightly linked with carotid atherosclerosis and therefore, this may be the pathophysiologic pathway underlying the undisputed association between anisocytosis and CV events [44,45]. In line with these results, RDW was reported as a strong risk marker for carotid atherosclerosis in patients with ischemic stroke [46] and hypertensive subjects [47]. Similar but limited data were reported in subjects with renal impairment: patients undergoing maintenance HD [48] and patients distributed in CKD stages 1–5 [9]. Recently, Khalil et al., showed that RDW was significantly associated with coronary calcium scores and the severity of coronary artery disease in a cohort of 100 T2DM patients [49]. Moreover, in this study a cut-off value above 14.6% (similar to the value we used to divide our population to high and low RDW groups) predicted the presence of severe coronary artery disease.

OS is present even at early stages of CKD, progresses along with disease severity and is further exacerbated by dialysis modalities [50,51]. Although it was reported that increased OS status results in erythrocytes fragility and a subsequent excessive rise of their heterogeneity, there is no data on the association between OS and RDW on CKD patients [52,53,54]. Since it was found that RDW was associated with the cholesterol content of RBC’s membranes in patients suffering angina [55] and OS is considered an inflammatory state of enhanced RBC fragility, we hypothesized that ox-LDL might be associated with RDW in our cohort. However, no association was found.

We found that RDW was closely linked with proteinuria, albuminuria and progression of DKD: diabetics in early CKD stages had higher RDW than subjects with normal kidney function, whereas RDW was gradually augmented along with CKD stages. Furthermore, RDW predicted progression of CKD diabetics to ESRD after 7 years of follow-up. The data on the association between RDW and renal impairment is limited. In line with our results, data from large epidemiologic studies showed a clear, graded and strong association between microalbuminuria and elevated RDW in general population [10]. In a population of patients with hypertension, RDW was an independent predictor of early renal impairment [56]. In the same study, after adjusted for age, gender and hemoglobin, albuminuria was an independent positive predictor of RDW levels. In agreement with our results, Lippi et al. [57], reported a strong, inverse, graded linkage between RDW and eGFR in a community based cohort of 8585 adults. After adjusted for gender, age, hemoglobin and MCV, presence of CKD (defined as eGFR < 60 mL/min) was a significant predictor of RDW levels. Data from the NHANES study [3], showed that compared to subjects in the lowest RDW quartile, those in the highest had a more than two-fold risk for CKD (5.2% versus 10.6, *p* < 0.001). In keeping with our results, RDW was negatively correlated with eGFR CKD patients stages 1–5 [9] and kidney transplant recipients [58]. As CKD progressed, every 10 mL/min decrease in eGFR was associated with a 27% increase of RDW. Moreover, CKD stage 3 subjects had an approximately two-fold risk and patients with CKD 4 and 5 had a three times higher risk for increased RDW compared to subjects with eGFR above 60 mL/min (OR = 1.99, 95% CI = 1.27–3.13 *p* = 0.02 and OR = 3.33, 95% CI = 1.56–7.14 *p* < 0.001 respectively). A recent study showed that elevated RDW not only was associated with kidney function at time of admission to intensive care unit, but also predicted onset of AKI and mortality [59]. Furthermore, anisocytosis was associated with increased risk of T2DM development in middle/old aged general population subjects [60]. The predictive value of RDW for DKD was stressed in a study by Magri et al. in a cohort of 196 T2DM patients with diabetic retinopathy [61]. Although no association between RDW and PAD, diabetic neuropathy was observed, RDW was a significant and independent predictor of DKD (OR 1.64, 95% CI 1.15–2.35, *p* = 0.006). Recently, Chen et al., reported that high RDW was independently associated with an increased risk of glomerulonephritis in T2DM patients with albuminuria and thus might serve as a novel predictive marker for differentiating glomerulonephritis and diabetic nephropathy in these patients [62]. The exact pathophysiologic mechanisms underlying the association between RDW and adverse outcomes in CKD and T2DM patients have not yet been fully elucidated. However, several mechanisms have been suggested to explain how an increase in RDW might serve as prognostic marker in these patients. Thus, it is hypothesized that elevated RDW might reflect several conditions associated with CKD that predispose to CV disease and death, including impaired glycemic control, persistent systemic inflammation, enhanced OS, impaired iron metabolism, hemodynamic overload changes, chronic tissue hypoxia, accelerated endothelial dysfunction and malnutrition [38]. All these mechanisms might explain why RDW is a novel, reliable prognostic marker in CKD and T2DM patients, suggesting that RDW might reflect several pathophysiologic processes.

In this study, we found an important inverse correlation between hemoglobin level and RDW value. Since the relationship between RDW and all-cause CV mortality persisted after adjusting for hemoglobin, it is fair to hypothesize that the association between RDW and this adverse event is independent of anemia status. According to our knowledge, this is the first study assessing the relation of RDW with deterioration of renal function and CV mortality in DKD. The major strengths of our study include the large cohort of patients with certified DKD divided in all 5 CKD stages, the long period of follow-up and the comprehensive data on our patients. Moreover, this is the first study, according to our knowledge that investigated the relation between RDW and oxidative stress -assessed by ox-LDL levels- in a graded cohort of DKD in all CKD stages. However, our study has certain limitations that should be addressed. Firstly, due to its retrospective design, no cause- effect relationship could be established. Secondly, we could not draw any concrete conclusions about the underlying mechanisms for our associations. Thirdly, the single-center design of this study does not allow generalization of our results. The small sample size should also be recognized as a limitation and although we adjusted for several confounding factors, there is a certain possibility that other important risk factors are not assessed in our study. Finally, since this is a cross-sectional study, no causal relationship can be established and our results should be interpreted with caution

Despite the accumulating data supporting the strong prognostic value of RDW, still is has not become clear whether anisocytosis reflects the cause of these pathological conditions or it is a simple epiphenomenon of underlying disorders such as inflammation, hypertension, hyperglycemia, enhanced oxidative stress, renal impairment or a combination of all the above. Future, larger, multicenter studies are needed in order to elucidate this aspect. Since it is an inexpensive, simple parameter available in the routine blood count, it might be assessed in everyday clinical practice for risk assessment in subjects with renal impairment and diabetes.

## 4. Materials and Methods

### 4.1. Patients

This retrospective cohort study was carried out at the Diabetic Kidney Disease Clinic of the University General Hospital of Alexandroupolis (Greece), using data from regular follow-up visits, electronic medical records and integrated telephone interviews for a period of seven years, from 20 November 2008 to 21 November 2015. Eligible patients for this study were those who had a recorded history of T2DM for at least 10 years. A total of 142 patients were included in our study, divided into two groups: 104 T2DM patients with established DKD, distributed in all stages of CKD-including stable patients undergoing maintenance HD and 38 (stage 0) with T2DM for more than 10 years, and normal fundoscopy, persistent normoalbuminuria and eGFR) above 60 mL/min. DKD was defined as eGFR < 60 mL/min, presence of diabetic retinopathy and albuminuria in three consecutive measurements in morning spot urine sample during a 6-month period. The diagnosis and classification of CKD stages were based on the National Kidney Foundation Kidney Disease Outcomes Quality Initiative criteria [63]. We estimated eGFR at baseline and at the end of this study using the CKD epidemiology collaboration (CKD-EPI) equation [64]. All HD patients had residual renal function. EGFR was calculated as eGFR from 24 h urine collection and blood (based on CKD-EPI calculation) divided by two. Exclusion criteria were clinical or laboratory evidence of non-diabetic nephropathy, acute illness and urinary tract disease. At baseline, all patients’ demographic, clinical and laboratory data were recorded, including documented history for previous CV event, assessment of ox-LDL) and measurement of cIMT. The end-points of our study were all-cause and CV mortality, fatal and non-fatal CV events and progression to ESRD. CV events were defined as coronary artery disease, stroke, angina, heart failure or Peripheral Artery Disease (PAD), documented by trained medical doctor after patients’ hospitalization. All participants were followed from enrollment until death or the study end, whichever came first. Patients provided written informed consent at baseline. The study protocol was approved by the Ethics Committee of the Scientific Council of the University General Hospital of Alexandroupolis with ethical approval code (1130/25 November 2011) and was in accordance with the Helsinki Declaration of Human Rights.

### 4.2. Laboratory Analyses

Fasting blood was drawn from all subjects into tubes with and without EDTA to obtain serum, plasma and whole blood. Samples for urea, creatinine, lipid parameters, CRP, HBA1c, were immediately transferred to the laboratory and assayed, while for oxidized LDL, samples were immediately centrifuged and plasma was stored at −20 °C, until analysis. Blood samples were collected from HD patients after an overnight 8 h fasting, immediately before the start of a dialysis session. Presence of proteinuria (UPCR) and albuminuria (UACR-Urinary Albumin to Creatinine Ratio), were defined in two out of three consecutive measurements in spot urine samples, during a 6 month period. Plasma ox-LDL levels were quantified by Enzyme-Linked Immunosorbent Assay (ELISA) according to the manufacturer’s instructions (human ox-LDL ELISA kit, Mercodia, Uppsala Sweden), as described elsewhere [65]. Detection limits for ox-LDL assays were established at 0.3 U/L, while intra- and interassay coefficients of variations were <10%. RDW measurement was performed by the automatic hematology analyzer Sysmex XE-5000 (Sysmex Corporation, Kobe, Japan), as part of the routine complete blood cell count. The reference range for RDW was 12.0–14.0% in our laboratory. To assess the predictive value of RDW on study outcomes, all patients were divided into two groups according to the median RDW value as: the high RDW group (>14.6%) and the low RDW group (≤14.6%).

### 4.3. CIMT Measurement

CIMT was measured by a B-mode, high-resolution, real time ultrasonography (ATL Ultrasound HDI 1300, Phillips, Bothell, WA, USA), by a single well-trained physician, as described before [66].

### 4.4. Statistics

All analyses were performed using the IBM Statistical Package for Social Sciences (SPSS 18.0 for Windows, IBM, Chicago, IL, USA). We calculated the sample size required to achieve a power of 80% in our study and a 5% level of significance. Estimating a 33% occurrence of the mortality outcome over 7 years in our cohort, as described in a recent study in a population similar to ours [39], we calculated a sample size of at least 136 patients to give approximately 80% power (alpha = 0.05, two-tailed) to reject the null hypothesis. We tested data for normality with Kolmogorov-Smirnov test. Continuous variables with normal distribution were presented as mean (standard deviation), whereas non-normally distributed continuous variables were presented as median (range). Patients’ characteristics were compared among groups of RDW using chi-square test for categorical variables and Mann-Whitney test for continuous variables. Spearman’s correlation co-efficient were used to examine bivariate associations between variables. To assess the significance of cIMT as a determinant of RDW value, we conducted a multiple regression analysis with RDW as the dependent variable in the absence and presence of possible predictors as determined in our correlation bivariate analyses. Non-normally distributed variables were log-transformed in the regression analyses.

Survival analyses were performed with Kaplan-Meier curves and log-rank tests. Cox proportional hazard analysis was used to evaluate adjusted HRs and 95% CIs for the associations between RDW and study outcomes, including all-cause and CV-related mortality, CV event and progression to ESRD. We performed Kaplan-Meier plots for all the study outcomes, stratified by CKD stage (Supplementary Figures S1–S4). To assess the possible association of various cofounders with our end-points, well-known risk factors such as age, sex, BMI, history of previous CV event, current tobacco smoking, duration of T2DM, total cholesterol, triglycerides, CRP, hemoglobin, HbA1c, UACR, eGFR, ox-LDL were tested in a regression Cox model. All variables associated or marginally lost association (*p* < 0.06) were included in the multivariate analyses. Significance was set at *p* < 0.05.

## Figures and Tables

**Table 1:** Anthropometric, clinical and biochemical parameters of patients with type 2 diabetes in the absence or presence of nephropathy above and below median RDW. Results for continuous variables are presented as mean (S.D.) or median (range).

	All Patients (*n* = 142)	RDW < 14.6% (*n* = 69)	RDW ≥ 14.6% (*n* = 73)	*p*
RDW (%)	14.6 (12.0–21.5)	13.4 (12.0–14.4)	15.8 (14.6–21.5)	<0.0001
Hemoglobin (g/dL)	12.38 ± 1.84	13.11 ± 1.55	11.69 ± 1.83	<0.0001
Age (years)	68.04 ± 9.05	68.33 ± 9.39	67.77 ± 8.77	0.85
Gender, Female (%)	45.8	49.23	50.77	0.51
Duration of HP (years)	14.56 ± 7.51	14.57 ± 7.81	14.56 ± 7.26	0.78
Duration of T2DM (years)	25.26 ± 7.83	13.81 ± 7.70	16.65 ± 7.76	0.013
Current smoking (%)	21.13	21.74	20.55	0.51
Presence of MI (%)	32.39	30.43	34.24	0.38
Presence of Stroke (%)	12.70	8.70	16.44	0.13
Presence of Angina (%)	26.76	26.09	27.40	0.51
Presence of PAD (%)	44.36	39.13	49.32	0.15
Presence of CVD (%)	65.5	60.87	69.87	0.17
SBP (mmHg)	137.32 ± 17.50	135.93 ± 18.57	138.68 ± 16.40	0.33
DBP (mmHg)	77.29 ± 8.89	76.49 ± 8.88	78.06 ± 8.89	0.16
BMI (kg/m^2^)	30.71 ± 5.22	29.99 ± 4.84	31.39 ± 5.50	0.06
Albumin (g/dL)	4.15 ± 0.45	4.22 ± 0.39	4.09 ± 0.48	0.13
Total cholesterol (mg/dL)	179.41 ± 52.48	179.87 ± 50.85	178.99 ± 54.33	0.76
LDL-cholesterol (mg/dL)	101.83 ± 43.94	101.59 ± 39.93	102.06 ± 47.80	0.81
HDL-cholesterol (mg/dL)	45.65 ± 12.69	46.40 ± 12.11	44.94 ± 13.25	0.34
Triglycerides (mg/dL)	139.0 (27.0–966.0)	149.72 (27.0–363.0)	170.67 (59.0–966.0)	0.61
HbA1c (%)	7.54 ± 1.14	7.61 ± 1.20	7.47 ± 1.10	0.94
CRP (mg/dL)	0.24 (0.0–14.0)	0.20 (0.0–11.0)	0.31 (0.0–14.0)	0.016
Urea (mg/dL)	86.36 ± 57.13	66.87 ± 47.90	104.78 ± 59.29	<0.0001
Creatinine (mg/dL)	2.48 ± 2.34	1.92 ± 1.99	3.03 ± 2.52	<0.0001
eGFR (mL/min/1.73 m^2^)	47.6 ± 31.81	57.84 ± 31.09	37.92 ± 29.54	<0.0001
UPCR	0.20 (0.01–9.70)	0.13 (0.01–5.50)	0.46 (0.01–9.70)	0.001
UACR (mg/g)	45.0 (1.0–9700.0)	26.00 (1.0–1800.0)	86.00 (2.4–9700.0)	0.001
cIMT (mm)	0.86 (0.40–1.78)	0.8 (0.40–1.61)	0.90 (0.40–1.78)	0.004
Ox-LDL (U/L)	61.07 (22.01–123.44)	64.65 (21.04–123.44)	59.91 (22.89–96.69)	0.21

*p* values of Kruskal-Wallis test or chi-square test for differences of variables among groups. HP, hypertension, T2DM, Type 2 diabetes mellitus; MI, myocardial infarction; PAD, peripheral artery disease; CVD, cardiovascular disease; SBP, systolic blood pressure; DBP, diastolic blood pressure; BMI, body mass index; LDL, low-density lipoprotein; HDL, high-density lipoprotein; HbA1c, glycated hemoglobin A1c; CRP, C-reactive protein; eGFR, estimated glomerular filtration rate; UPCR, urinary protein-creatinine ratio; UACR, urinary albumin-creatinine ratio; cIMT, carotid intima-media thickness; Ox-LDL, oxidized low-density lipoprotein.

**Table 2:** Correlations of various parameters with RDW.

Parameters	*r*	*p*
Anthropometric—Clinical Parameters		
Age (years)	−0.05	0.58
Duration of HP (years)	0.02	0.82
Duration of T2DM (years)	0.19	0.023
SBP (mmHg)	0.15	0.09
DBP (mmHg)	0.18	0.03
BMI (kg/m^2^)	0.16	0.052
Hematological Parameters		
Hemoglobin (g/dL)	−0.41	<0.0001
Nutritional—Inflammatory Parameters		
Albumin (g/dL)	−0.20	0.026
Total cholesterol (mg/dL)	−0.03	0.76
Triglycerides (mg/dL)	0.09	0.27
Ox-LDL (U/L)	−0.90	0.35
HbA1c (%)	0.08	0.37
CRP (mg/dL)	0.24	0.004
DKD Markers		
Urea (mg/dL)	0.38	<0.0001
Creatinine (mg/dL)	0.32	<0.0001
eGFR (mL/min/1.73 m^2^)	−0.33	<0.0001
UPCR	0.30	0.001
UACR (mg/g)	0.29	0.002
Vascular Calcification Markers		
cIMT (mm)	0.22	0.008

Spearman’s rho test and Mann-Whitney correlation. HP, hypertension, T2DM, Type 2 diabetes mellitus; SBP, systolic blood pressure; DBP, diastolic blood pressure; BMI, body mass index; Ox-LDL, oxidized low-density lipoprotein; HbA1c, glycated hemoglobin A1c; CRP, C-reactive protein; eGFR, estimated glomerular filtration rate; UACR, urinary albumin-creatinine ratio; cIMT, carotid intima-media thickness

**Table 3:** Multiple regression analysis (stepwise forward) in 142 type 2 diabetes patients with cΙΜΤ as the dependent variable.

	β	SE	*p*	CI
Model 1 (Unadjusted)				
Age	0.008	0.03	0.004	0.003–0.013
Model 2 (Adjusted) ^a^				
Age	0.008	0.03	0.001	0.003–0.013
RDW	0.031	0.012	0.012	0.007–0.056
BMI	0.009	0.004	0.042	0.0–0.018

^a^ Adjusted for age, sex, diastolic/systolic blood pressure, body mass index, duration of type 2 diabetes mellitus, triglycerides, total cholesterol, glycated hemoglobin A1c, C-reactive protein, estimated glomerular filtration rate, urinary albumin-creatinine ratio, red blood cell distribution width. SE, standard error; CI, confidence interval.

**Table 4:** Multiple regression analysis (stepwise forward) in 142 type 2 diabetes patients with RDW as the dependent variable.

	β	SE	*p*	CI
Model 1 (Unadjusted)				
Hemoglobin	−0.44	0.10	<0.001	−0.65 to −0.24
Model 2 (Adjusted) ^a^				
Hemoglobin	−0.38	0.10	<0.001	−0.58 to −0.18
UPCR	0.30	0.10	0.003	0.10–0.49
cIMT	1.59	0.64	0.014	0.33–2.86

^a^ Adjusted for hemoglobin, urinary protein to creatinine ratio, carotid intima media thickness, diastolic blood pressure, duration of type 2 diabetes mellitus, serum albumin, C-reactive protein, estimated glomerular filtration rate. SE, standard error; CI, confidence interval.

**Table 5:** Cox proportional hazard analysis (forward stepwise regression) showing predictors for all-cause mortality, cardiovascular mortality, fatal/non-fatal CV events and progression to ESRD in multivariate models in patients with high and low RDW levels.

	*B*	*HR*	*95% CI*	*p*
All-Cause Mortality				
RDW	0.24	1.27	1.1–1.61	0.04
eGFR	−0.03	0.96	0.94–0.99	0.02
History of CV event	2.33	10.27	1.32–80.1	0.03
Cardiovascular Mortality				
RDW	0.26	1.30	1.1–1.56	0.01
eGFR	−0.03	0.97	0.94–0.99	0.009
Age	−0.08	1.1	1.01–1.16	0.02
History of CV event	1.40	4.0	1.1–14.77	0.04
Cardiovascular Events				
RDW	0.33	1.40	1.17–1.66	<0.0001
History of CV event	1.95	7.00	2.69–18.21	<0.0001
Progression to ESRD				
RDW	0.31	1.36	1.11–1.66	0.003
eGFR	−0.05	0.95	0.92–0.98	0.001
BMI	−0.13	0.88	0.80–0.98	0.02

Model for all-cause mortality adjusted for age, sex, history of previous CV event, duration of T2DM, CRP, hemoglobin, UACR, eGFR. Model for CV mortality adjusted for age, sex, history of previous CV event, duration of T2DM, hemoglobin, UACR, eGFR. Model for CV events adjusted for age, sex, history of previous CV event, duration of T2DM, CRP, hemoglobin, UACR, eGFR. Model for progression to ESRD adjusted for age, sex, BMI, duration of T2DM, CRP, hemoglobin, UACR, eGFR. HR, hazard ratio; CI, confidence interval.

## Supplementary Materials

The following are available online at https://www.mdpi.com/2075-1729/10/11/301/s1, Figure S1: All-cause mortality in (A) Controls, (B) CKD stages1–4 and (C) Hemodialysis. Figure S2: Cardiovascular mortality in (A) Controls, (B) CKD stages1–4 and (C) Hemodialysis. Figure S3. Cardiovascular events in (A) Controls, (B) CKD stages1–4 and (C) Hemodialysis. Figure S4. Progression to ESRD in (A) Controls, (B) CKD stages1–4 and (C) Hemodialysis. Table S1: Multiple regression analysis (stepwise forward) in 142 type 2 diabetes patients with cΙΜΤ as the dependent variable, adjusting for HD. Table S2. Multiple regression analysis (stepwise forward) in 142 type 2 diabetes patients with RDW as the dependent variable, adjusting for HD.
