# Supplementary material for: Red Blood Cell Distribution Width Is Associated with Deterioration of Renal Function and Cardiovascular Morbidity and Mortality in Patients with Diabetic Kidney Disease
Source: Life (Basel). 2020 Nov 22;10(11):301. doi: 10.3390/life10110301 (PMC7700598; doi:10.3390/life10110301)

life

Supplementary Files

Red Blood Cell Distribution Width is Associated with Deterioration of Renal Function and Cardiovascular Morbidity and Mortality in Patients with Diabetic Kidney Disease

Stefanos Roumeliotis [1,*], Aikaterini Stamou [2], Athanasios Roumeliotis [1], Marios Theodoridis [3], Konstantinos Leivaditis [1], Stylianos Panagoutsos [3] and Vassilios Liakopoulos [1]

Supplementary Tables

Table S1. Multiple regression analysis (stepwise forward) in 142 type 2 diabetes patients with cIMT as the dependent variable, adjusting for HD.

	β	SE	p	CI
**Model 1 (unadjusted)**				
Age	0.008	0.03	**0.006**	0.002–0.013
**Model 2 (adjusted)[a]**				
Age	0.008	0.03	**0.003**	0.003–0.014
RDW	0.029	0.013	**0.029**	0.003–0.054
BMI	0.010	0.005	**0.031**	0.001–0.02

[a]Adjusted for age, sex, diastolic/systolic blood pressure, Body Mass Index, duration of type 2 diabetes mellitus, triglycerides, total cholesterol, Glycated Hemoglobin A1c, C-Reactive Protein, Estimated Glomerular Filtration Rate, Urinary Albumin-Creatinine Ratio, Red Blood Cell Distribution Width. SE, Standard Error; CI, Confidence Interval.

Table S2. Multiple regression analysis (stepwise forward) in 142 type 2 diabetes patients with RDW as the dependent variable, adjusting for HD.

	β	SE	p	CI
**Model 1 (unadjusted)**				
Hb	–0.46	0.10	**<0.001**	–0.67 to –0.26
**Model 2 (adjusted)[a]**				
Hb	–0.40	0.10	**<0.001**	–0.60 to –0.19
UPCR	0.27	0.10	**0.011**	0.06–0.47
cIMT	1.54	0.64	**0.019**	0.26–2.82

[a]Adjusted for hemoglobin, urinary protein to creatinine ratio, carotid intima media thickness, diastolic blood pressure, duration of type 2 diabetes mellitus, Serum Albumin, C-Reactive Protein, Estimated Glomerular Filtration Rate. SE, Standard Error; CI, Confidence Interval.

Supplementary Figures

Figure S1. All-cause mortality in (A) Controls, (B) CKD stages1-4 and (C) Hemodialysis.

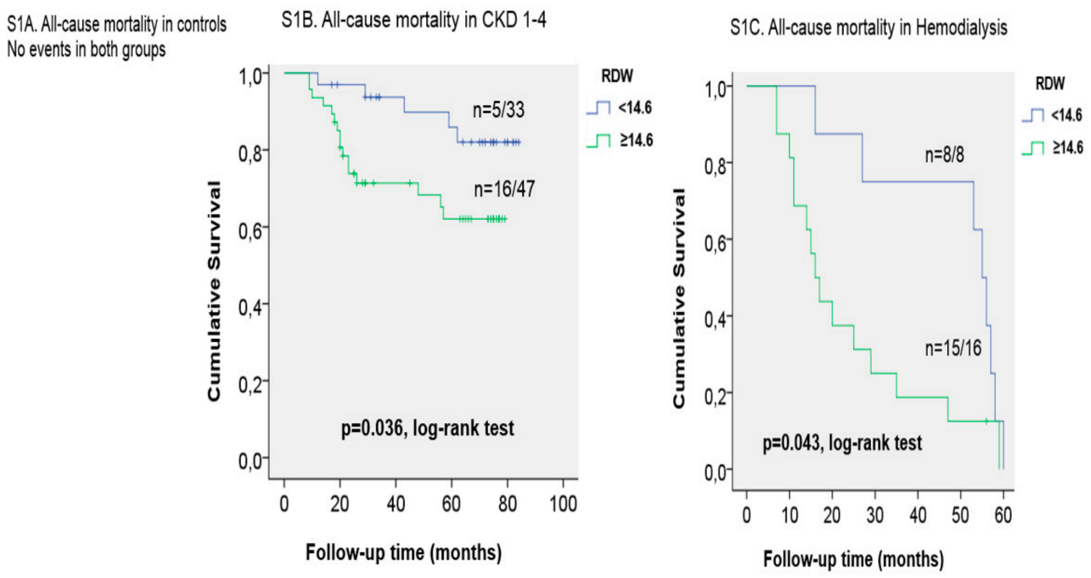

Figure S2. Cardiovascular mortality in (A) Controls, (B) CKD stages1-4 and (C) Hemodialysis.

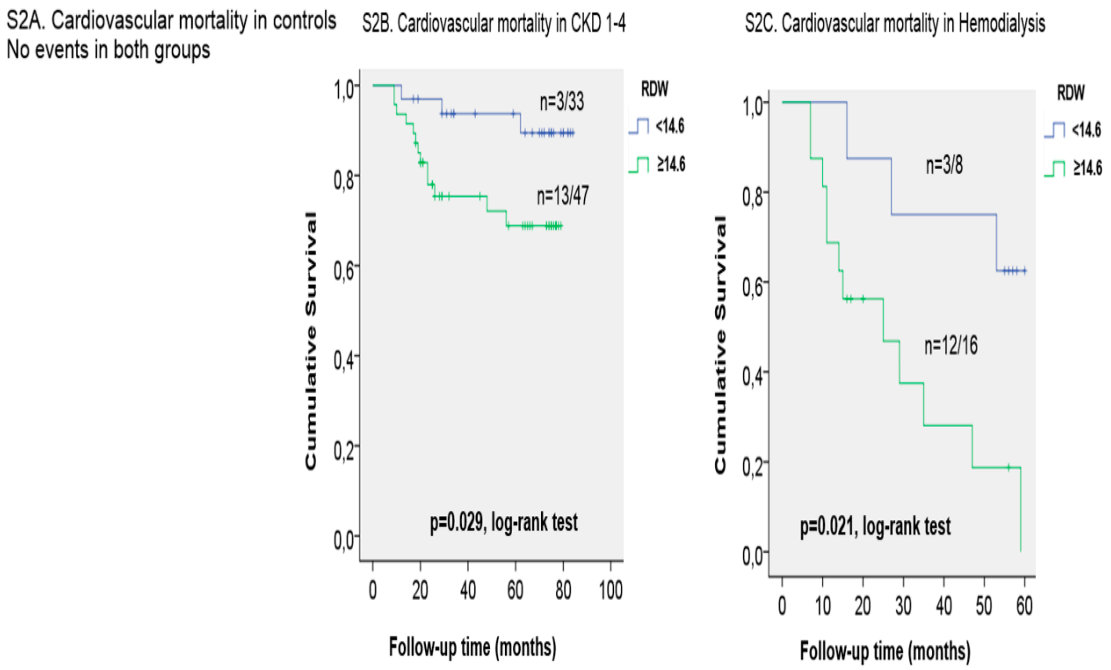

Figure S3. Cardiovascular events in (A) Controls, (B) CKD stages1-4 and (C) Hemodialysis.

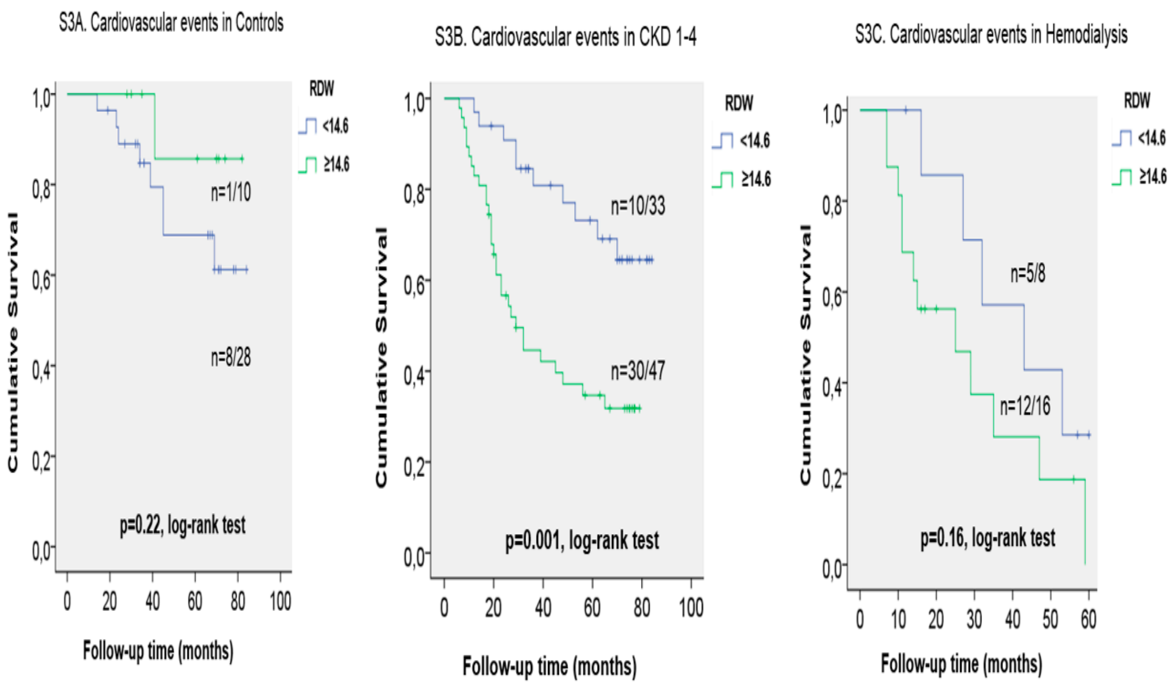

Figure S4. Progression to ESRD in (A) Controls, (B) CKD stages1-4 and (C) Hemodialysis.

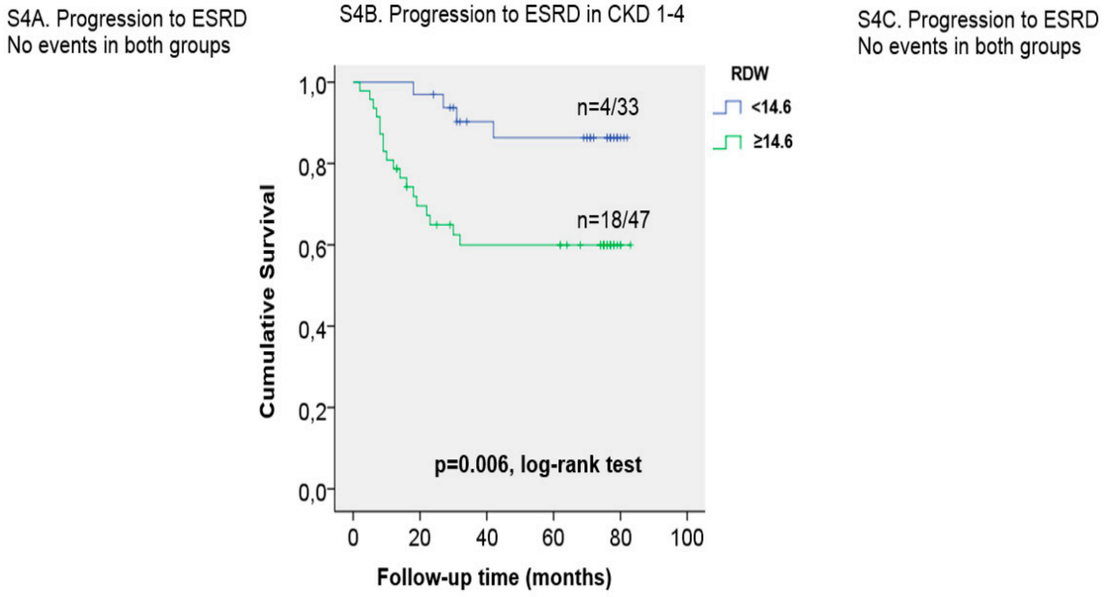

Supplement: Supplementary file 1 [file life-10-00301-s001.pdf]
